# Association of urine mitochondrial DNA with clinical measures of COPD in the SPIROMICS cohort

**DOI:** 10.1172/jci.insight.181925

**Published:** 2024-06-24

**Authors:** William Z. Zhang, Michelle C. Rice, Katherine L. Hoffman, Clara Oromendia, Igor Z. Barjaktarevic, J. Michael Wells, Annette T. Hastie, Wassim W. Labaki, Christopher B. Cooper, Alejandro P. Comellas, Gerard J. Criner, Jerry A. Krishnan, Robert Paine, Nadia N. Hansel, Russell P. Bowler, R. Graham Barr, Stephen P. Peters, Prescott G. Woodruff, Jeffrey L. Curtis, Meilan K. Han, Karla V. Ballman, Fernando J. Martinez, Augustine M.K. Choi, Kiichi Nakahira, Suzanne M. Cloonan, Mary E. Choi, the SPIROMICS Investigators

Original citation *JCI Insight*. 2020;5(3):e133984. https://doi.org/10.1172/jci.insight.133984

Citation for this corrigendum: *JCI Insight*. 2024;9(12):e181925. https://doi.org/10.1172/jci.insight.181925

A recent reassessment of consent forms from the SPIROMICS I patient cohort enrolled from 2010 to 2016 determined that 8 participants (1 never smoker, 2 smokers without airflow obstruction, 3 mild/moderate COPD, and 2 severe COPD) included in the *JCI Insight* paper did not provide appropriate consent for inclusion in genetic studies. Thus, the analyses that included these data have been redone to exclude these individuals. The figures, tables, text, and supplemental data have been corrected to reflect the updated analyses. The authors have stated that the updated analyses do not alter the conclusions of the study. A sentence in the results section, “U-mtDNA associates with different clinical outcomes in males and females,” has been corrected in to better reflect the updated analysis. The corrected sentence appears below:

Additionally, there were nominal associations between u-mtDNA and radiographic measures of emphysema among males in models that adjusted for age, BMI, and smoking status.

Sample numbers, *P* values, and u-mtDNA levels have been updated in online HTML and PDF versions of the manuscript.

The authors regret the errors.

## Version 1 06/24/2024

Electronic publication

